# Predictors of impaired awareness of hypoglycaemia and severe hypoglycaemia in adults with type 1 diabetes

**DOI:** 10.1111/dme.70074

**Published:** 2025-05-19

**Authors:** Nicola N. Zammitt, Shareen Forbes, Berit Inkster, Mark W. J. Strachan, Rohana J. Wright, Anna R. Dover, Roland H. Stimson, Fraser W. Gibb

**Affiliations:** ^1^ Edinburgh Centre for Endocrinology & Diabetes NHS Lothian Edinburgh UK; ^2^ University/BHF Centre for Cardiovascular Science, Queen's Medical Research Institute University of Edinburgh Edinburgh UK

**Keywords:** hypoglycaemia, severe hypoglycaemia, type 1 diabetes

## Abstract

**Aims:**

This study aimed to assess the prevalence of impaired awareness of hypoglycaemia (IAH) and severe hypoglycaemia (SH) in adults with type 1 diabetes and identify risk factors for both conditions in a contemporary cohort.

**Methods:**

A cross‐sectional survey was conducted on 782 adults with type 1 diabetes. Participants completed a questionnaire including validated hypoglycaemia awareness and mental health tools. Continuous glucose monitoring (CGM) data were collected in 402 participants. SH was identified based on self‐reported episodes.

**Results:**

89% were CGM users and 27% were using continuous subcutaneous insulin infusion (CSII). 5.3% of participants reported a recent episode of SH and 21% had IAH based on the Gold score. Elevated Gold Score was independently associated with socioeconomic deprivation (OR 1.9, *p* = 0.002), female sex (OR 1.8, *p* = 0.002) and positive depression screen (OR 2.1, *p* = 0.007). Hypoglycaemia detection threshold <3.0 mM was independently associated with older age (OR 1.03 per year, *p* < 0.001) and positive depression screen (OR 2.7, *p* < 0.001). Greater glucose variability (OR 1.14 per % CV glucose, *p* < 0.001), positive anxiety screen (OR 3.0, *p* = 0.031) and detection threshold <3.0 mM (OR 6.7, *p* < 0.001) were all independently associated with SH risk.

**Conclusions:**

The prevalence of SH is lower in the modern era of type 1 diabetes management and may reflect greater use of CGM and CSII. Mental health symptoms and socioeconomic deprivation are key associations with IAH and SH. Risk models incorporating clinical, psychological and CGM data may more effectively predict SH.

**What's new?**

**What is already known?**

- Estimates of severe hypoglycaemia (SH) rates in type 1 diabetes vary substantially and often relate to an earlier era in glucose management strategies. Predictors of SH have also largely been assessed in a prior era.

**What this study has found?**

- SH rates were much lower than previously reported.
- Socioeconomic deprivation, mental health symptoms and CGM‐derived glucose variability were associated with SH risk.

**What are the implications of the study?**

- Clinicians should consider these associations when assessing SH risk and be aware of the limitations in tools used to assess SH risk.

## 1 INTRODUCTION

Hypoglycaemia has been a significant complication in the treatment of diabetes since the first use of insulin therapy and remains a key barrier to achieving optimal glycaemic targets.^1^ Severe hypoglycaemia (SH) or level 3 hypoglycaemia is generally accepted to represent an episode where a person with diabetes requires the help of another person to treat a low blood glucose event.^2^, ^3^ SH is associated with high levels of distress, both in people living with diabetes and their family members.^4^ SH has also been associated with increased cardiovascular risk and mortality.^5^ Identifying those at elevated risk for SH is, therefore, extremely important. Failure to recognise hypoglycaemia at glucose concentration <3.0 mM is associated with a fourfold increase risk of SH.^6^ The Gold score^7^ is another common metric used to detect impaired awareness of hypoglycaemia (IAH) and is associated with significantly higher rates of SH.^8^ IAH is said to affect up to 30% of people with type 1 diabetes^9^ and historical rates of SH have been reported as high as 40% in those with a long duration of type 1 diabetes.^10^ Clearly the treatment landscape in type 1 diabetes has transformed over the past decade, including therapies such as CGM and CSII, which are associated with reduced rates of hypoglycaemia (including SH).^11^, ^12^ We sought to determine the prevalence of IAH and SH, and risk factors for these, in a contemporary cohort of adults with type 1 diabetes.

## 2 METHODS

This was a cross‐sectional, ‘real‐world’ assessment of all adults (age >18 years) with type 1 diabetes attending the Royal Infirmary of Edinburgh diabetes clinic (United Kingdom). As a service evaluation of routinely collected data this project did not require ethical approval. All 2210 individuals were sent a copy of our standard clinic questionnaire (supplementary materials) in October 2022, to facilitate patient‐centred improvements in service delivery. 782 people (35%) returned a completed questionnaire (by post or online). The questionnaire covered hypoglycaemia awareness, severe hypoglycaemia, mental health screening (GAD‐2 and PSQ‐2^13^) and smoking status. Specifically, IAH was assessed by Gold score^7^ and an analogue scale of the glucose level at which people typically experience symptoms of hypoglycaemia. Clinical and demographic data were obtained from SCI‐Diabetes (Scottish national diabetes register). CGM data (all Freestyle Libre 2) was obtained from LibreView (14‐day data capture). To be included, CGM data had to correspond to the date of questionnaire response and a minimum of 70% data capture had to be available. CGM data satisfying these criteria were available in 402 people (51% of all questionnaire respondents). Key CGM metrics are reported in line with international consensus guidance.^14^

Data are presented as median (IQR). Unpaired data were compared by the Wilcoxon rank‐sum test. Categorical data were compared by *χ* ^2^ test. Logistic regression analysis assessed independent predictors of IAH and SH. *p* Values <0.05 were considered statistically significant. Statistical analyses were performed using R Studio (version 2023.12.1).

## 3 RESULTS

### 3.1 Cohort characteristics and generalisability

Demographic and clinical characteristic of the cohort are presented in Table 1. Compared to those who did not respond to the questionnaire, the cohort is older, with longer diabetes duration, lower HbA1c, lower socioeconomic deprivation and higher use of diabetes technology. 5.3% reported an episode of SH since their last clinic consultation (typical clinic follow‐up interval 6–12 months). 21% had IAH based on a Gold score ≥4 and 10% reported a typical hypoglycaemia detection threshold below 3.0 mM. SH was reported in 3.0% of individuals with a Gold score <4 and in 12% of those with a Gold score ≥4 (*p* < 0.001). Similarly, SH was reported in 4.0% of those with a hypoglycaemia detection threshold ≥3.0 mM and in 16% of those with a threshold <3.0 mM (*p* < 0.001). 49% of people reporting SH had a Gold score <4 and 70% had a hypoglycaemia detection threshold >3.0 mM. Gold score had a sensitivity of 51% and specificity of 80% for classifying SH risk. Awareness threshold below 3.0 mM had a sensitivity of 30% and specificity of 91% for classifying SH risk.

**TABLE 1:** Characteristics of those included in this cohort compared with individuals who did not complete the questionnaire (CGM data were available in 402/782 of those who completed the questionnaire).

	Included in cohort (*N* = 782)	Not included in cohort (*N* = 1428)	*p*
Age (years)	51 (36–63)	43 (29–58)	<0.001
Diabetes duration (years)	24 (13–37)	20 (11–32)	<0.001
Sex	Male 54% Female 46%	Male 57% Female 43%	0.291
SIMD quintile (1 – most deprived and 5 least deprived)	1 8.1% 2 18% 3 16% 4 21% 5 37%	1 13% 2 24% 3 19% 4 18% 5 26%	<0.001
BMI (kg/m^2^)	27.1 (23.9–30.5)	25.9 (22.9–29.8)	<0.001
HbA1c (mmol/mol)	60 (52–69)	66 (57–79)	<0.001
GAD‐2 anxiety screen positive	17%	NA	
PSQ‐2 depression screen positive	19%	NA	
Current smoker	8.7%	NA	
CGM user	89%	69%	<0.001
CSII user	27%	18%	<0.001
Average glucose (mM)	9.8 (8.5–11.5)	NA	
GMI (mmol/mol)	59 (53–67)	NA	
CV glucose (%)	34.6 (30.4–38.7)	NA	
TBR (%)	1 (0–3)	NA	
TIR (%)	53 (37–69)	NA	
TAR (%)	44 (28–61)	NA	
Severe hypoglycaemia	5.3%	NA	
IAH gold (≥4)	21%	NA	
IAH threshold	10%	NA	

Abbreviations: BMI, body mass index; CGM, continuous glucose monitoring; CSII, continuous subcutaneous insulin infusion; CV glucose, coefficient of variation of glucose; GAD‐2, Generalised Anxiety Disorder 2‐item questionnaire; GMI, glucose management indicator; HbA1c, glycated haemoglobin; IAH, impaired awareness of hypoglycaemia; PHQ‐2, Patient Health Questionnaire 2‐item depression screen; SIMD, Scottish Index of Multiple Deprivation; TAR, time above range (glucose >10.0 mmol/L); TBR, time below range (glucose <3.9 mmol/L); TIR, time in range (glucose 3.9–10.0 mmol/L).

### 3.2 Gold score

**FIGURE 1:**
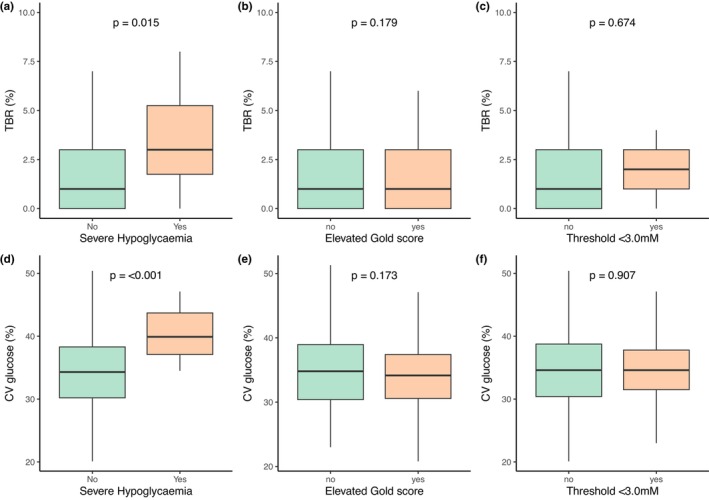
TBR and CV glucose compared by SH, IAH Gold and IAH threshold status.

IAH, by Gold score, was significantly higher in cigarette smokers and those with greater socioeconomic deprivation (Table 2). Positive anxiety (GAD‐2) and depression (PSQ‐2) screen results were twofold higher in people with IAH (Table 2). Women were 50% more likely to have an elevated Gold score than men (*p* = 0.002). Age, diabetes duration, HbA1c, C‐peptide status, BMI, technology use and CGM metrics (Figure 1) were not significantly different between those with and without IAH, based on Gold score (Table 2). Logistic regression analysis identified socioeconomic deprivation (OR 1.9 for those in quintiles 1 and 2 [95% CI 1.3–2.8], *p* = 0.002), female sex (OR 1.8 [95% CI 1.2–2.6], *p* = 0.002) and positive depression screen (OR 2.1 [95% CI 1.2–3.7], *p* = 0.007) as independently associated with Gold score ≥4, with reference to those with Gold Score <4.

**TABLE 2:** Comparison of clinical and demographic features between those with normal hypoglycaemia awareness and impaired awareness as defined by Gold score ≥4 (CGM data were available in 319/601 with no IAH Gold and 80/163 with IAH Gold).

	No IAH Gold (*n* = 601)	IAH Gold (*n* = 163)	*p*
Age (years)	51 (36–61)	53 (36–65)	0.211
Diabetes duration (years)	24 (14–36)	23 (9–38)	0.382
Sex	Male 58% Female 42%	Male 44% Female 56%	0.002
SIMD rank (out of 6976 – 1 is most deprived)	4959 (2942–6390)	3704 (2201–5685)	<0.001
BMI (kg/m^2^)	27.1 (24.0–30.6)	27.1 (23.2–30.4)	0.294
HbA1c (mmol/mol)	60 (52–68)	63 (52–73)	0.086
GAD‐2 anxiety screen	Positive 14% Negative 86%	Positive 28% Negative 72%	<0.001
PSQ‐2 depression screen	Positive 15% Negative 85%	Positive 32% Negative 68%	<0.001
Smoking status	Smoker 14% Non‐smoker 86%	Smoker 7% Non‐smoker 93%	0.010
CGM user	Yes 89% No 11%	Yes 89% No 11%	0.982
CSII user	Yes 28% No 72%	Yes 26% No 74%	0.829
Average glucose (mM)	9.8 (8.4–11.5)	9.7 (8.8–11.6)	0.280
GMI (mmol/mol)	59 (52–67)	58 (54–67)	0.288
CV glucose (%)	34.8 (30.4–39.0)	34.2 (30.6–37.4)	0.173
TBR (%)	1 (0–4)	1 (0–3)	0.179
TIR (%)	53 (38–70)	53 (36–66)	0.347
TAR (%)	44 (27–61)	46 (30–64)	0.257
Severe hypoglycaemia	Yes 3.0% No 97%	Yes 12% No 88%	<0.001

Abbreviations: BMI, body mass index; CGM, continuous glucose monitoring; CSII, continuous subcutaneous insulin infusion; CV glucose, coefficient of variation of glucose; GAD‐2, Generalised Anxiety Disorder 2‐item questionnaire; GMI, glucose management indicator; HbA1c, glycated haemoglobin; PHQ‐2, Patient Health Questionnaire 2‐item depression screen; SIMD, Scottish Index of Multiple Deprivation; TAR, time above range (glucose >10.0 mmol/L); TBR, time below range (glucose <3.9 mmol/L); TIR, time in range (glucose 3.9–10.0 mmol/L).

### 3.3 Hypoglycaemia detection threshold

IAH, based on a hypoglycaemia detection threshold <3.0 mM, was significantly higher in older individuals and those with greater socioeconomic deprivation (Table 3). A positive depression screen (PSQ‐2) but not an anxiety screen (GAD‐2) was associated with threshold‐based IAH (Table 3). Diabetes duration, sex, BMI, HbA1c, C‐peptide status, smoking, technology use and CGM metrics (Figure 1) were not associated with IAH, based on the hypoglycaemia detection threshold (Table 3). Logistic regression identified older age (OR 1.03 per year [95% CI 1.01–1.04], *p* < 0.001) and a positive depression screen (OR 2.7 [95% CI 1.6–4.7], *p* < 0.001) as independently associated with IAH based on the hypoglycaemia detection threshold; SIMD rank (socioeconomic deprivation) was not (*p* = 0.090), with reference to those with a hypoglycaemia detection threshold ≥3.0 mM.

**TABLE 3:** Comparison of clinical and demographic features between those with normal hypoglycaemia awareness and impaired awareness as defined by hypoglycaemia detection threshold <3.0 mM (CGM data were available in 363/704 with no IAH threshold and 39/78 with IAH threshold).

	No IAH threshold (*n* = 704)	IAH threshold (*n* = 78)	*p*
Age (years)	50 (35–62)	57 (46–68)	0.002
Diabetes duration (years)	23 (13–37)	28 (16–39)	0.248
Sex	Male 55% Female 45%	Male 49% Female 51%	0.364
SIMD rank (out of 6976 – 1 is most deprived)	4922 (2742–6323)	3788 (2312–5564)	0.005
BMI (kg/m^2^)	27.1 (23.9–30.5)	27.0 (24.5–29.8)	0.943
HbA1c (mmol/mol)	60 (52–69)	62 (52–71)	0.196
GAD‐2 anxiety screen	Positive 16% Negative 84%	Positive 23% Negative 77%	0.133
PSQ‐2 depression screen	Positive 17% Negative 83%	Positive 35% Negative 65%	<0.001
Smoking status	Smoker 8.7% Non‐smoker 91%	Smoker 9.2% Non‐smoker 91%	0.871
CGM user	Yes 89% No 11%	Yes 86% No 14%	0.542
CSII user	Yes 28% No 72%	Yes 21% No 79%	0.221
Average glucose (mM)	9.8 (8.5–11.5)	9.6 (8.7–11.9)	0.678
GMI (mmol/mol)	59 (53–67)	58 (54–69)	0.678
CV glucose (%)	34.6 (30.4–38.8)	34.6 (31.5–37.8)	0.907
TBR (%)	1 (0–4)	2 (1–3)	0.674
TIR (%)	53 (37–68)	54 (37–71)	0.926
TAR (%)	44 (28–62)	42 (28–61)	0.872
Severe hypoglycaemia	Yes 4.0% No 96%	Yes 16% No 84%	<0.001

Abbreviations: BMI, body mass index; CGM, continuous glucose monitoring; CSII, continuous subcutaneous insulin infusion; CV glucose, coefficient of variation of glucose; GAD‐2, Generalised Anxiety Disorder 2‐item questionnaire; GMI, glucose management indicator; HbA1c, glycated haemoglobin; PHQ‐2, Patient Health Questionnaire 2‐item depression screen; SIMD, Scottish Index of Multiple Deprivation; TAR, time above range (glucose >10.0 mmol/L); TBR, time below range (glucose <3.9 mmol/L); TIR, time in range (glucose 3.9–10.0 mmol/L).

### 3.4 Severe hypoglycaemia (level 3 hypoglycaemia)

History of SH was associated with socioeconomic deprivation and with positive depression (PSQ‐2) and anxiety (GAD‐2) screening (Table 4). In addition, SH was associated with higher TBR and greater CV glucose (Figure 1). Time below 3.0 mM was also significantly higher in those with SH (0 [0–1] vs. 0 [0–0], *p* = 0.031). Logistic regression identified positive anxiety screen (OR 3.0 [95% CI 1.4–6.2], *p* = 0.004), hypoglycaemia detection threshold below 3.0 mM (OR 2.6 [95% CI 1.0–5.9], *p* = 0.032) and high Gold score (OR 2.9 [95% CI 1.4–6.1], *p* = 0.005) as independent predictors of SH, with reference to those with no history of SH. In a model incorporating CGM data, CV glucose (OR 1.14 [95% CI 1.07–1.23], *p* < 0.001), positive anxiety screen (OR 3.0 [95% CI 1.05–8.02], *p* = 0.031) and hypoglycaemia detection threshold below 3.0 mM (OR 6.7 [95% CI 2.4–18.0], *p* < 0.001) were independent predictors of SH with reference to those with no history of SH (full regression models are presented in Data S1).

**TABLE 4:** Comparison of clinical and demographic features between those with no recent history of SH and those reporting recent SH (CGM data were available in 385/741 with no SH and 17/40 with SH).

	No SH (*n* = 741)	SH (*n* = 40)	*p*
Age (years)	51 (36–63)	48 (33–60)	0.531
Diabetes duration (years)	24 (13–37)	24 (16–38)	0.368
Sex	Male 54% Female 46%	Male 50% Female 50%	0.692
SIMD rank (out of 6976–1 is most deprived)	4840 (2780–6310)	4180 (2021–5505)	0.023
BMI (kg/m^2^)	27.1 (23.9–30.5)	26.6 (22.6–30.3)	0.548
HbA1c (mmol/mol)	60 (52–69)	61 (51–75)	0.463
GAD‐2 anxiety screen	Positive 16% Negative 84%	Positive 41% Negative 59%	<0.001
PSQ‐2 depression screen	Positive 18% Negative 82%	Positive 42% Negative 58%	<0.001
Smoking status	Smoker 8.6% Non‐smoker 91%	Smoker 11% Non‐smoker 89%	0.871
CGM user	Yes 89% No 11%	Yes 88% No 12%	0.800
CSII user	Yes 27% No 73%	Yes 20% No 80%	0.409
Average glucose (mM)	9.8 (8.5–11.5)	9.7 (8.6–12.9)	0.451
GMI (mmol/mol)	59 (53–67)	58 (53–73)	0.471
CV glucose (%)	34.3 (30.2–38.3)	39.9 (37.1–43.7)	<0.001
TBR (%)	1 (0–3)	3 (2–6)	0.015
TIR (%)	53 (37–69)	51 (32–68)	0.430
TAR (%)	44 (28–61)	42 (28–68)	0.620

Abbreviations: BMI, body mass index; CGM, continuous glucose monitoring; CSII, continuous subcutaneous insulin infusion; CV glucose, coefficient of variation of glucose; GAD‐2, Generalised Anxiety Disorder 2‐item questionnaire; GMI, glucose management indicator; HbA1c, glycated haemoglobin; PHQ‐2, Patient Health Questionnaire 2‐item depression screen; SIMD, Scottish Index of Multiple Deprivation; TAR, time above range (glucose >10.0 mmol/L); TBR, time below range (glucose <3.9 mmol/L); TIR, time in range (glucose 3.9–10.0 mmol/L).

## 4 DISCUSSION

We have shown that SH is likely to be much less common in the current era of type 1 diabetes management. This is perhaps unsurprising in the context of very high levels of CGM (89%) and CSII (27%) use; both therapies have been associated with a substantial reduction in severe hospitalised hypoglycaemia in Scotland (25% for CGM and 33% for CSII).^11^, ^12^ In addition, we have identified very strong relationships between mental health and socioeconomic deprivation with respect to both IAH and SH. We did not find any association between CGM metrics and IAH but we did see significantly elevated time below range and glucose variability in those reporting recent SH.

It seems highly likely that the incidence of SH has fallen substantially from historic values cited in the literature (including annual rates of 40% in adults with long duration of type 1 diabetes).^10^ Differences in the precise definition of SH, the timescale in which episodes are captured (e.g. within 3 months or 6 months etc.) and heterogeneity between cohorts can make comparison between studies difficult. Data captured between 2016 and 2018 from the T1D Exchange Registry (USA) reported a 6% rate of SH in the preceding 3 months (with lower rates in CSII users who comprised 63% of the cohort)^15^; this figure is not hugely different from the 5.3% reported in our cohort. Similarly, Norwegian registry data (capturing almost 50% of adults with T1 diabetes in the country) reported an annual SH rate of 7.3%.^16^ In contrast, a recent US‐based, single‐centre study reported a 26% rate of SH in the preceding 6 months^17^ and recent T1D Exchange data reported an annual rate of 20% in a cohort of over 2000 people, with high levels of CGM (92%) and automated insulin delivery (51%) use.^18^ The marked difference between rates in our cohort (and the large Norwegian cohort) and those reported in the United States perhaps relates to differences in enrolment to the study (potentially greater bias to participate in those with active SH issues) or the structure of health care provision (perhaps those with greater IAH/SH issues were likelier to be referred for specialist care in T1D Exchange centres). The response rate of 64% cited in the large Norwegian cohort,^16^ with high levels of CGM and CSII use, is impressive and is likely to make this the most representative published data on modern SH incidence.

Current methods for classifying SH risk, such as Gold score and glucose threshold for symptoms, have reasonable specificity but very low sensitivity for identifying people at risk. Almost half of those reporting SH had normal awareness based on Gold score. This may be an inevitable consequence of the inherent unpredictability of SH; however, it raises the possibility that more sophisticated models, incorporating risk scores, clinical data and CGM metrics could perform more effectively.

These data highlight how common mental health symptoms are in people with type 1 diabetes, and consideration should be given to systematic screening to help direct people to effective interventions. Our findings with respect to anxiety and depression symptoms are striking as, unlike some other associations, they extend across both IAH and SH risk. Our findings are consistent with previous reports that such symptoms are associated with IAH.^19^ Although it is not possible to infer causation, it is feasible to speculate that a bi‐directional relationship exists. IAH and SH are recognised to generate substantial distress^1^ and could exert a negative influence upon mental health symptoms. It is also conceivable that commonalities between symptoms of anxiety and hypoglycaemia could generate delays in early identification of hypoglycaemia.^19^ Given the links between mental health, IAH and SH risk, it is perhaps not surprising that demand for automated insulin delivery was highest in those with positive GAD‐2 and PSQ‐2 screening scores.^20^ In addition to technology, evidence‐based educational and behavioural approaches to reduce hypoglycaemia and diabetes distress are available.^21^, ^22^

Socioeconomic deprivation was associated with a 2.6‐fold increase in hospitalised severe hypoglycaemia in Scotland (when comparing the least and most deprived quintiles).^23^ We have shown a strong relationship not only with SH but also with IAH. Those with greatest socioeconomic deprivation are also least likely to achieve target HbA1c levels^24^ and are least likely to use modern diabetes technology^20^; these data reinforce the need to address glaring inequalities in outcome and treatment provision in type 1 diabetes.

This study represents one of the largest assessments of comprehensive CGM data in relation to IAH and SH. Consistent with the findings of Choudhary et al.^25^ we did not find any significant difference in CGM metrics between those with IAH and normal hypoglycaemia awareness. We found higher TBR and CV glucose in those with SH, but no difference in average glucose and high glucose metrics. A small study (*n* = 100) assessing CGM identified higher CV glucose and average glucose (but not TBR) in those with SH. This study used a range of different CGM systems, and rates of SH were substantially higher than we observed, making direct comparison difficult.^17^ However, the common finding (and the most striking difference in our cohort) was the association with glucose variability. Intuitively, high levels of glucose variability should equate to increased SH risk and this has now been confirmed in two separate cohorts. Most recently, data from the ABCD Freestyle Libre Audit (*n* = 5029) found significant associations between TBR and both IAH and SH, although the magnitude of the association is relatively modest.^26^ Interestingly, the proportion of people with higher than recommended TBR (≥4%) was greater in the ABCD audit (58%) than in our cohort (25%). Despite this, self‐reported SH rates were even lower in the ABCD audit (4%) than in our cohort. IAH, established with the five‐item HypoA‐Q IA tool, was independently associated with time in CGM‐detected level 1 and level 2 hypoglycaemia (but not CV glucose) in 804 adults with type 1 diabetes.^27^ This highlights that the modality used to define IAH may have a significant influence upon the relationship with CGM metrics.

A key strength of this study was the size of cohort with detailed demographic and clinical data. In particular, the subgroup of individuals with comprehensive CGM data is one of the largest studied in the context of SH and IAH. The uniform use of a single CGM system may also be an advantage given clinically relevant variability between sensor types.^28^ This is also one of the largest assessments of IAH and SH risk in the modern era, with very high rates of CGM use. Compared to our total clinic population, those included in this analysis are older, more affluent and had lower HbA1c. However, very few studies assessing IAH appear to be truly representative of the wider type 1 diabetes population and are frequently more skewed with respect to low HbA1c and high levels of CSII use.^15^, ^17^ As a real‐world assessment, utilising our routine clinic questionnaire, there is a lack of precision with respect to the timeframe in which SH episodes are recorded (‘since last clinic visit’ rather than a specific number of months) and we do not have detailed data on the number of SH events. As is the case with all ascertainment of SH, some individuals may be reluctant to disclose this information, particularly in relation to its potential impact on driving entitlement.^29^ However, this is likely to apply to all recent studies and the trend towards substantially lower SH rates appears consistent, at least in European cohorts.^16^, ^26^

We have shown that SH is likely to be less common than prior historical data suggests in the modern era of type 1 diabetes management. We have also shown that socioeconomic deprivation, mental health symptoms and glucose variability are important associations with SH and should be considered when assessing risk alongside IAH. As automated insulin delivery becomes established as the standard of care in type 1 diabetes, rates of SH and associated risk factors are likely to change and will become a key focus for future research.

## CONFLICT OF INTEREST STATEMENT

No relevant disclosures.

## Supporting information


Data S1.

## Data Availability

The data that support the findings of this study are available on request from the corresponding author. The data are not publicly available due to privacy or ethical restrictions.
