# Supplementary material for: Predictors of impaired awareness of hypoglycaemia and severe hypoglycaemia in adults with type 1 diabetes
Source: Diabet Med. 2025 May 19;42(10):e70074. doi: 10.1111/dme.70074 (PMC12434435; doi:10.1111/dme.70074)
Supplement: Supplementary file 1 — Data S1. [file DME-42-e70074-s001.docx]

**Supplementary materials**

**Questionnaire**


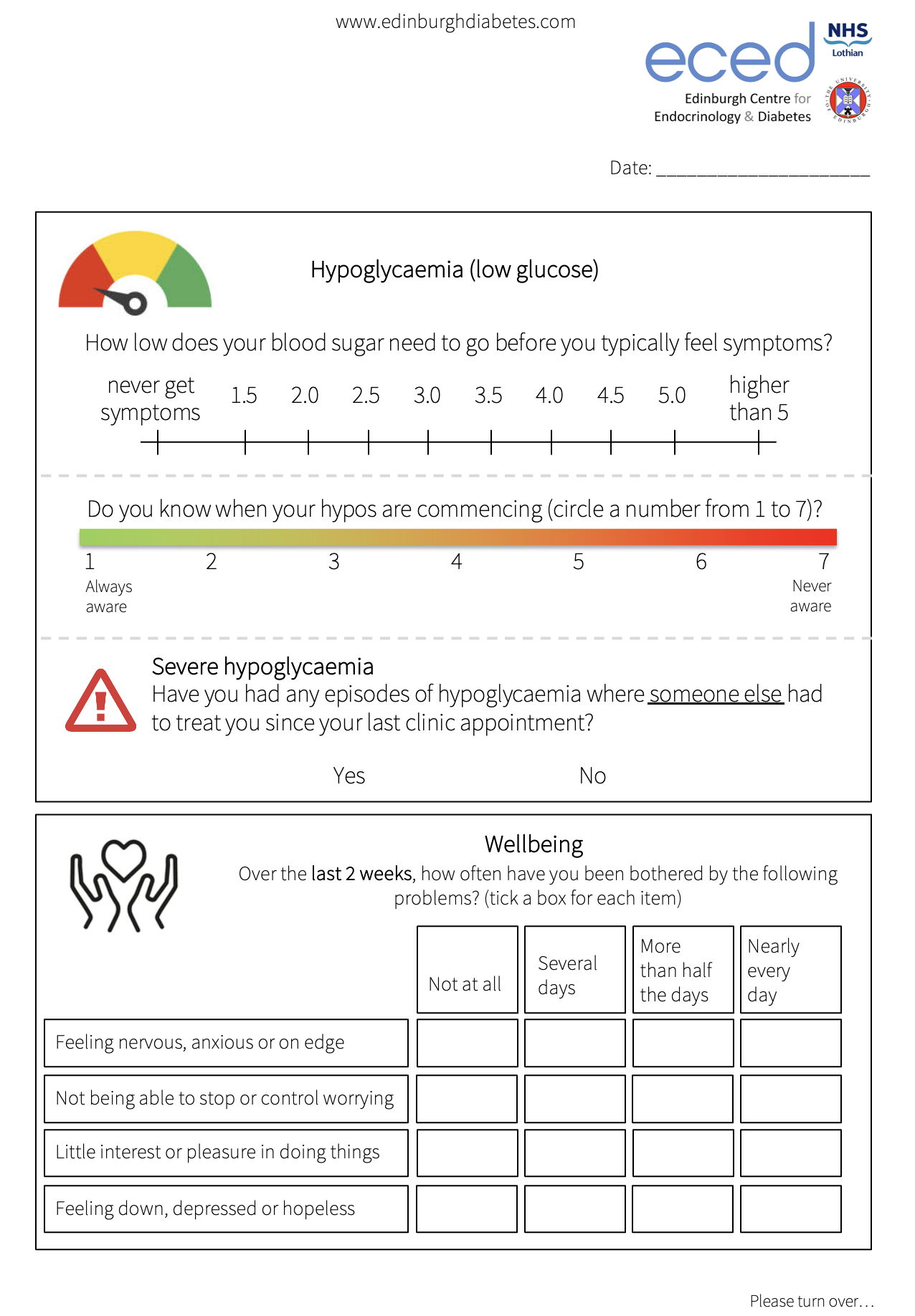


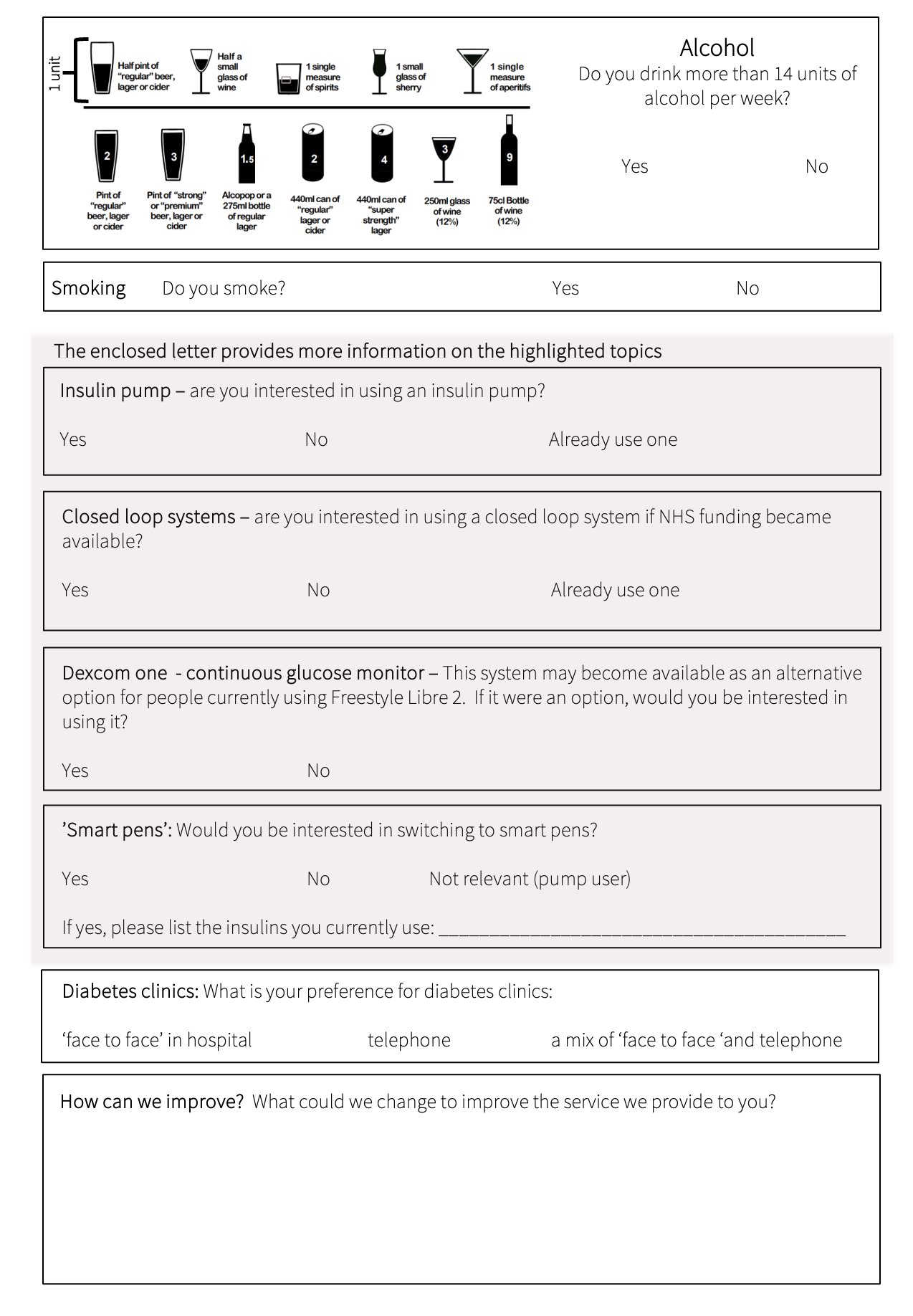


**Logistic regression models**

Logistic regression model for IAH (Gold):

| **Variable** | **Odds Ratio (OR)** | **95% Confidence Interval** | **p-value** |
| --- | --- | --- | --- |
| **Intercept** | 0.42 | 0.28 – 0.61 | <0.001 |
| Anxiety (screen positive) | 1.54 | 0.86 – 2.71 | 0.140 |
| Depression (screen positive) | 2.15 | 1.22 – 3.74 | 0.007 |
| SIMD quintile (3–5 vs 1–2) | 0.54 | 0.36 – 0.80 | 0.002 |
| Gender (Male vs Female) | 0.56 | 0.38 – 0.81 | 0.002 |

Logistic regression model for IAH (glucose threshold):

| **Variable** | **Odds Ratio (OR)** | **95% Confidence Interval** | **p-value** |
| --- | --- | --- | --- |
| **Intercept** | 0.03 | 0.01 – 0.09 | <0.001 |
| Depression (positive) | 2.73 | 1.57 – 4.67 | <0.001 |
| Age (per year increase) | 1.03 | 1.01 – 1.04 | <0.001 |
| SIMD rank (continuous) | 1.00 | 1.00 – 1.00 | 0.090 |

Logistic regression model for SH (not including CGM data):

| **Variable** | **Odds Ratio (OR)** | **95% Confidence Interval** | **p-value** |
| --- | --- | --- | --- |
| **Intercept** | 0.02 | 0.01 – 0.04 | <0.001 |
| Anxiety (screen positive) | 2.95 | 1.38 – 6.16 | 0.004 |
| Threshold < 3 mmol/L | 2.57 | 1.03 – 5.91 | 0.032 |
| Gold IAH | 2.89 | 1.35 – 6.10 | 0.005 |

Logistic regression model for SH (including CGM data):

| **Variable** | **Odds Ratio (OR)** | **95% Confidence Interval** | **p-value** |
| --- | --- | --- | --- |
| **Intercept** | 0.00018 | <0.00001 – 0.003 | <0.001 |
| Anxiety (screen positive) | 3.00 | 1.05 – 8.02 | 0.032 |
| Hypoglycaemia < 3 mmol/L | 6.75 | 2.43 – 18.00 | <0.001 |
| Coefficient of variation (CV) glucose | 1.14 | 1.07 – 1.23 | <0.001 |
